# Determinant of catastrophic costs associated with treatment for rifampicin-resistant TB in households in the Republic of Moldova

**DOI:** 10.5588/ijtldopen.23.0608

**Published:** 2024-06-01

**Authors:** A. Ciobanu, V. Plesca, S. Doltu, M. Manea, L. Domente, A. Dadu

**Affiliations:** ^1^ University of Medicine and Pharmacy ‘Nicolae Testemitanu’, Chisinau, Republic of Moldova;; 2 WHO Regional Office for Europe, Copenhagen, Denmark;; 3 Institute of Phthisiopneumology ‘Chiril Draganiuc’, Chisinau, Republic of Moldova;; 4 National Agency of Public Health, Chisinau, Republic of Moldova;; 5 Act for Involvement, Chisinau, Republic of Moldova;; 6 Independent Consultant, Chisinau, Republic of Moldova

**Keywords:** RR-TB, household income, out-of-pocket payment, poverty, Moldova, tuberculosis

## Abstract

**SETTING:**

The Republic of Moldova is a lower-middle-income country. Patients with TB face some barriers to accessing TB services. Welfare benefits are available during TB treatment.

**OBJECTIVES:**

We aimed to determine the proportion of rifampicin-resistant TB (RR-TB) households that experienced catastrophic costs due to TB at a threshold of ≥20% of household income and investigate the associated risk factors.

**DESIGN:**

A cross-sectional countrywide study comprised 430 patients with RR-TB who had received TB treatment as an inpatient or outpatient for at least 2 months.

**RESULTS:**

RR-TB patients lost 30% of their household income in inpatient and 70% in outpatient TB care. TB-related costs were associated with being unofficially employed or unemployed (aOR 1.9, 95% CI 1.1–3.3), having fewer household members (aOR 2.1, 95% CI 1.3–3.5), having an income that accounted for over 50% of household income (aOR 2.4, 95% CI 1.5–3.8), and being a poor household (aOR 2.2, 95% CI 1.2–3.9).

**CONCLUSION:**

Although TB health services are provided to patients free of charge, 26% of RR-TB households experienced catastrophic TB costs. The associated factors should be considered to improve patient-centred TB care, especially in vulnerable groups. Welfare payments mitigate TB costs.

## RÉSUMÉ

**CADRE:**

La République de Moldova est un pays à revenu intermédiaire de la tranche inférieure. Les patients atteints de TB se heurtent à certains obstacles pour accéder aux services de lutte contre la TB. Des prestations sociales sont disponibles pendant le traitement de la TB.

**OBJECTIFS:**

Nous avons cherché à déterminer la proportion de ménages atteints de TB résistant à la rifampicine (RR-TB) qui ont subi des coûts catastrophiques dus à la TB à un seuil de ≥20% du revenu du ménage et à étudier les facteurs de risque associés.

**MÉTHODE:**

Une étude transversale à l'échelle nationale a porté sur 430 patients atteints de RR-TB qui avaient reçu un traitement antituberculeux en hospitalisation ou en consultation externe pendant au moins 2 mois.

**RÉSULTATS:**

Les patients atteints de RR-TB ont perdu 30% du revenu de leur ménage en hospitalisation et 70% en soins ambulatoires. Les coûts liés à la TB étaient associés au fait d'avoir un emploi non officiel ou un chômeur (OR ajusté [ORa] 1,9 ; IC à 95% 1,1 à 3,3), d'avoir moins de membres du ménage (ORa 2,1 ; IC à 95% 1,3 à 3,5), d'avoir un revenu représentant plus de 50 % du revenu du ménage (ORa 2,4 ; IC à 95% 1,5 à 3,8) et d'être un ménage pauvre (ORa 2,2 ; IC à 95% 1,2 à 3,9).

**CONCLUSION:**

Bien que les services de santé liés à la TB soient fournis gratuitement aux patients, 26% des ménages atteints de RR-TB ont subi des coûts catastrophiques. Les facteurs associés doivent être pris en compte pour améliorer les soins de la TB centrés sur le patient, en particulier dans les groupes vulnérables. Les prestations sociales atténuent les coûts de la TB.

According to the Sustainable Development Goals (SDGs), achieving the goal of universal health coverage (UHC) would ensure that everyone receives the health services they need, that public health services are of sufficient quality to be effective, and that accessing these services does not expose users to financial hardship.^1^ UHC and eliminating catastrophic costs incurred by households affected by TB are key objectives of the WHO’s End TB Strategy 2015–2035.^2^ Catastrophic costs due to TB are defined by the WHO as total costs exceeding 20% of household annual income.^3^

Poverty is an enabler of the TB burden and has a significant economic impact.^4^ The Republic of Moldova is a lower-middle-income country where the poverty headcount ratio at national poverty lines is 23% of the population, and 44% of current health expenditure is paid out of pocket.^5,6^ The country ranks third in the WHO European Region for countries with the highest numbers of families on the brink of poverty and who need to pay out of pocket to access health services.^7^

The Republic of Moldova is one of the 30 countries with the highest levels of rifampicin-resistant TB (RR-TB).^8^ The key impact indicators, TB mortality and TB notification rate, decreased by respectively 66% (from 18 to 6 per 100,000) and 38% (from 113 to 72 per 100 000) from 2010 to 2019. The latest achievements reported by the National TB Programme (NTP) for RR-TB include the detection of 73% of pulmonary RR-TB cases (WHO 2018 estimate) and the successful treatment of RR-TB of 53% (WHO’s European Region target is 75%).^9^

The NTP objectives stipulate universal access to TB services for diagnosis and treatment.^10^ However, patients incur costs while accessing TB services.^3^ In this study, we aimed to determine the proportion of households experiencing catastrophic costs due to RR-TB; to investigate the determinants, and assess differences between poor and non-poor households in terms of TB-related costs. The study findings should provide authorities with a snapshot of the costs faced by households when accessing health care for TB services. Moreover, this study will inform social and financial protection solutions and interventions to be defined that should be targeted at households affected by TB.

## METHODS

### Study design

This cross-sectional study of patients with RR-TB was conducted from September 2016 to January 2017.

### General setting

The Republic of Moldova is situated in southeast Europe with a population of approximately 3 million inhabitants (including approximately 0.5 million inhabitants in the breakaway region of the Left Bank of the Dniester). The gross national income is US$3,930 per capita.^11^

### National TB Control Programme

The Ministry of Health has overall responsibility for TB control in the country, undertaking this function through the ‘Chiril Draganiuc’ Institute of Phthisiopneumology, Chisinau, Republic of Moldova, in collaboration with other governmental entities, civil society organisations and international partners. TB services are provided through a network of specialised TB facilities and primary health care. TB treatment is performed in both inpatient and outpatient settings. There is a system of welfare benefits for all TB patients, such as temporary disability allowances for those who are officially employed and adherence incentives (Table 1).

**Table 1.** Definitions used in the operational research.

Term	Definitions/comments
Indirect cost	Indirect cost was estimated based on self-reported income and considered income in monetary terms reported by the respondents during the interviews in terms of both their own income and that of their households
	We used cost per day and number of treatment days to estimate indirect cost in inpatient and outpatient care: cost per day in inpatient care was assessed based on the average monthly income self-reported by the respondent for the period prior to TB; cost per day in outpatient care was assessed based on the difference between the average monthly income self-reported by the respondent for the period prior to TB and the income earned by the patient during the outpatient treatment
Direct cost	Direct costs were defined as medical and non-medical expenses
	Direct medical costs comprised formal and informal payments claimed by respondents to have been incurred for TB services from the time of diagnosis until the completion of treatment and included payments for tests, laboratory investigations, consultations, non-traditional medicine, TB drugs or other pharmaceuticals required to deal with side effects or comorbidities
	Direct non-medical costs comprised payments incurred in relation to accessing and/or benefiting from medical services from the time of diagnosis until the completion of treatment, such as foodstuffs, hygiene items, or personal items in the inpatient stage and travel costs to TB sites for medical visits or directly observed treatment visits; travel costs were calculated based on the distance travelled to TB sites* and the number of journeys
Total household income	Total household income was estimated based on household income and welfare transfers
	Household income was estimated based on self-reported income by respondents for their household for the period prior to TB and for inpatient and outpatient care
	Welfare transfers included temporary disability allowances and incentives provided to TB patients during outpatient care. The amount of temporary disability allowance was estimated by using the amount earmarked for TB that was applicable to officially employed respondents (monthly amount × number of months in treatment). The amount of temporary disability allowance payable to TB patients each month is equal to 100% of baseline value, i.e., the amount the patient usually receives from the employer. Temporary disability allowances were provided by social protection. Incentives (such as cash or food stamps) were supported by health insurance company, provided to keep a TB patient compliant with TB therapy in outpatient settings) and were calculated as follows:1) MDL315 per month × number of months in TB outpatient treatment (as applicable before 1 July 2015);2) MDL35 per day × number of days in TB outpatient treatment (as applicable after 1 July 2015);3) MDL35 per day × number of days in TB outpatient treatment (as applicable to patients initially starting non-MDR-TB treatment before initiating MDR-TB therapy)
Catastrophic cost	Total costs borne by patients (direct and indirect) related to TB, equal to or exceeding the threshold cut-off value of 20% of total household income
Wealth Index^18^	The Wealth Index is a composite measure of the cumulative living standard of a household. It is calculated using data on a household’s ownership of a selected set of assets, such as televisions, bicycles and cars; dwelling characteristics such as flooring material; type of drinking water source; and toilet and sanitation facilities. The Wealth Index considers characteristics related to wealth status, avoiding variables that do not represent an asset, or outcome variables
World Bank poverty criterion^17^	According to the World Bank criterion, a poor household is a household earning <US$1.9per capita per day

* Cost per journey.

MDL = Moldovan leu; MDR-TB = multidrug-resistant TB.

Only one-third of patients with TB have health insurance before being diagnosed with TB.^12^ The absence of health insurance can create barriers to accessing TB diagnostic services. The NTP follows the WHO guidelines for TB diagnosis and treatment. There is a national TB case-based database, Information System for Monitoring and Evaluation of TB patients (SIME TB),^12^ for TB patient notifications and follow-up.

### Study population

A list of potential participants was extracted from SIME TB, consecutively selected based on the following inclusion criteria: 1) new or relapse RR-TB case; 2) adult (≥18 years); 3) mentally competent to undergo a structured interview; 4) having received TB treatment for the intensive phase in a hospital setting for at least 2 months, or receiving TB treatment for the continuation phase in outpatient care for at least 2 months, or having completed treatment for at least 2 months prior to interview. Patients from prisons and the Left Bank of the Dniester River were excluded.

Based on the eligibility criteria, 450 patients with RR-TB were selected for interview, of whom 20 (4.4%) refused to be interviewed. The remaining 430 were included in the analysis.

#### Data collection, sources and variables

Study participants were contacted in person by trained interviewers and were interviewed after obtaining written informed consent. Three types of questionnaires (inpatient, outpatient and post-treatment) were developed based on the WHO tool for assessing TB-related costs.^3^ The questionnaires included questions on demographic and socioeconomic status, harmful habits, clinical features, TB-related costs, and income loss. Information on TB treatment history was extracted from the SIME TB database.

Participants who were interviewed in the intensive phase reported on pre-diagnostic, diagnostic, and treatment costs in hospital settings; those who were interviewed in the continuation phase and after treatment completion reported on treatment costs in ambulatory care.

Direct costs were considered medical and non-medical costs, and indirect costs were considered income loss (Table 1). To estimate the total treatment costs, we extrapolated the monthly reported costs in the intensive and continuation phases to all participants. To measure income loss, we established household income earned using the incomes of patients and family members, from wages, welfare benefits, adherence incentives, and other income, before TB diagnosis and during TB treatment. According to the World Bank criterion, a household earning less than US$1.9 per capita per day was classified as a poor household.^13^ Household income was categorised using the Wealth Index based on the Multiple Indicator Cluster Surveys methodology.^14^

Using the WHO definition, catastrophic health costs were defined as total costs of TB incurred by a household equal to or exceeding 20% of the household’s annual income.^3^ The costs were calculated in terms of Moldovan leu (MDL) and converted to US$ (exchange rate: US$1 = MDL19.9238). Details on costs (direct and indirect) and definitions of other variables are provided in Table 1.

### Data analysis

The survey data were double entered using MS Access 2003 (Microsoft, Redmond, WA, USA) and imported into IBM SPSS Statistics v20.0 (IBM Corp, Armonk, NY, USA) for analysis. Categorical variables were described as absolute numbers and frequencies, and continuous variables were presented as means (standard deviation [SD]) and medians (interquartile range [IQR]) as appropriate. Kruskal–Wallis, χ^2^ tests, and *Z*-tests were used to assess patient profiles and compare proportions, and *t*-tests were used for continuous variables. The Mann–Whitney *U*-test was used to compare median costs. The odds ratio (OR) was selected as a measure of association in the analysis of determinates. Using binary logistic regression, we estimated the significance (*P*-value), crude odds ratios (ORs), and confidence intervals (CIs). To identify the best model and estimate the significance, adjusted ORs, and 95% CIs of the determinants, we included all variables for which *P* < 0.1 in the univariate analysis and age and sex, disregarding their significance as common confounders in the multivariate analysis. The interaction between costs and selected covariates was measured consecutively so that the effect of determinants on catastrophic TB costs could vary depending on the value of other variables. The significance of the interactions was measured using the Wald test. The level of significance throughout the analysis was set at 5%.

### Ethical approval

The study protocol was approved by the National Committee for Ethical Expertise of Clinical Trials of the Republic of Moldova. Written informed consent was obtained from all participants.

## RESULTS

Our analysis included 430 patients (150 in the intensive phase, 137 in the early continuation phase and 143 in the late continuation phase). The median age of the study population was 42 years; 78% were male, and 55% lived in a poor household. Table 2 shows the baseline sociodemographic and clinical characteristics of patients with TB. The median annual household income before TB was US$1,857 (IQR 1,205–1,857), and the median payment for TB was US$417 (IQR 297–641).

**Table 2.** Sociodemographic and clinical characteristics of patients with rifampicin-resistant TB overall and stratified by type of TB care, Republic of Moldova, 2016.

Characteristic	Total	Inpatient	Outpatient	End of treatment	*P*-value^*^
	*n* (%)	*n* (%)	*n* (%)	*n* (%)	
Total, *n*	430	150	137	143	
Sex					0.977
Male	336 (78.1)	118 (78.7)	107 (78.1)	111 (77.6)	
Female	94 (21.9)	32 (21.3)	30 (21.9)	32 (22.4)	
Age, years, mean ± SD	41.7 ± 12.4	41.4 ± 11.8	42.1 ± 12.8	41.6 ± 12.8	0.899
Age, years					0.924
≤39	198 (46.0)	68 (45.3)	65 (47.4)	65 (45.5)	
≥40	232 (54.0)	82 (54.7)	72 (52.6)	78 (54.5)	
Residence					0.696
Urban	160 (37.2)	55 (36.7)	48 (35.0)	57 (39.9)	
Rural	270 (62.8)	95 (63.3)	89 (65.0)	86 (60.1)	
Level of education					0.204
None/primary	143 (33.3)	61 (40.7)	42 (30.7)	40 (28.0)	
Secondary	215 (50.0)	67 (44.7)	71 (51.8)	77 (53.8)	
Vocational/higher	72 (16.7)	22 (14.7)	24 (17.5)	26 (18.2)	
Marital status					0.95
Married/cohabitating	224 (52.1)	77 (51.3)	71 (51.8)	76 (53.1)	
Unmarried	206 (47.9)	73 (48.7)	66 (48.2)	67 (46.9)	
Household size, number of persons					0.271
1	195 (45.3)	65 (43.3)	63 (46.0)	67 (46.9)	
2–3	164 (38.1)	52 (34.7)	55 (40.1)	57 (39.9)	
≥4	71 (16.5)	33 (22.0)	19 (13.9)	19 (13.3)	
Employment					0.05
Official employed	162 (37.7)	72 (48.0)	45 (32.8)	45 (31.5)	
Unofficially employed or not employed	268 (62.3)	78 (52.0)	92 (67.2)	98 (68.5)	
Households in poverty^†^					0.571
Poor	238 (55.3)	79 (52.7)	75 (54.7)	84 (58.7)	
Non-poor	192 (44.7)	71 (47.3)	62 (45.3)	59 (41.3)	
Wealth index^‡^					0.715
Low standard of living	215 (50.0)	71 (47.3)	71 (51.8)	73 (51.0)	
High standard of living	215 (50.0)	79 (52.7)	66 (48.2)	70 (49.0)	
Health insurance status prior to TB					<0.001
Yes	226 (52.6)	55 (36.7)	73 (53.3)	98 (68.5)	
No	204 (47.4)	95 (63.3)	64 (46.7)	45 (31.5)	
History of migration					0.283
Yes	78 (18.1)	31 (20.7)	19 (13.9)	28 (19.6)	
No	352 (81.9)	119 (79.3)	118 (86.1)	115 (80.4)	
History of incarceration^§^					0.362
Yes	41 (9.5)	15 (10.4)	16 (12.0)	10 (7.0)	
No	378 (87.9)	129 (89.6)	117 (88.0)	132 (93.0)	
Current smoking status					0.255
Yes	246 (57.2)	93 (62.0)	78 (56.9)	75 (52.4)	
No	184 (42.8)	57 (38.0)	59 (43.1)	68 (47.6)	
Alcohol consumption					0.463
Yes	45 (10.5)	14 (9.3)	18 (13.1)	13 (9.1)	
No	385 (89.5)	136 (90.7)	119 (86.9)	130 (90.9)	
Injectable/non-injectable drugs use					0.049
Yes	43 (10.0)	22 (14.7)	12 (8.8)	9 (6.3)	
No	387 (90.0)	128 (85.3)	125 (91.2)	134 (93.7)	
History of TB treatment					0.013
New	289 (67.2)	110 (73.3)	79 (57.7)	100 (69.9)	
Relapse	141 (32.8)	40 (26.7)	58 (42.3)	43 (30.1)	
HIV					0.667
Yes	36 (8.4)	15 (10.0)	10 (7.3)	11 (7.7)	
No	394 (91.6)	135 (90.0)	127 (92.7)	132 (92.3)	
Diabetes mellitus					0.361
Yes	11 (2.6)	6 (4.0)	2 (1.5)	3 (2.1)	
No	419 (97.4)	144 (96.0)	135 (98.5)	140 (97.9)	
Comorbidities					<0.001
Yes	135 (31.4)	57 (38.0)	39 (28.5)	39 (27.3)	
No	295 (68.6)	93 (62.0)	98 (71.5)	104 (72.7)	
Side-effects					<0.001
Yes	352 (81.9)	141 (94.0)	103 (75.2)	108 (75.5)	
No	78 (18.1)	9 (6.0)	34 (24.8)	35 (24.5)	

* Calculated using the Kruskal–Wallis H-test.

† Poverty defined using the World Bank criterion and based on household income prior to TB diagnosis. Missing data were excluded during hypothesis testing.

‡ Categorized using the Wealth Index.

§ Data available from 419 patients.

SD = standard deviation.

The median TB-related costs in inpatient care did not differ between poor (US$245) and non-poor patients (US$228, *P* = 0.580). The highest payments made by TB patients in inpatient care were for nonmedical products (US$230 for poor patients, US$216 for non-poor patients, *P* = 0.659) and for food (US$180 poor patients, US$173 non-poor TB patients, *P* = 0.753) (Table 3).

**Table 3.** Direct and indirect costs related to TB stratified by income at household level (World Bank criterion) for patients with rifampicin-resistant TB, Republic of Moldova, 2016.*

	Total	Households in poverty^†^		*P*-value^‡^
	Median [IQR] US$	Poor	Non-poor	
		Median [IQR] US$	Median [IQR] US$	
Direct cost, total	416.8 [297.3–641.2]	379.3 [272.5–612.7]	458.4 [316.0–695.8]	0.02
Pre-diagnosis and diagnosis	5.0 [0.0–5.0]	5.0 [3.3–5.0]	5.0 [0.0–5.0]	0.367
Public clinics	3.3 [0.0–3.3]	3.3 [2.5–3.3]	3.3 [0.0–3.3]	0.296
Private clinics	1.2 [0.0–1.2]	1.2 [0.0–1.2]	1.2 [0.0–1.2]	0.861
Inpatient care	239.0 [139.5–316.8]	244.7 [149.6–311.5]	227.6 [126.0–317.0]	0.580
Medical costs:	10.0 [0.0–14.7]	10.1 [0.0–14.6]	9.6 [0.0–14.8]	0.938
Tests and medical examinations	0.2 [0.0–0.4]	0.2 [0.0–0.4]	0.2 [0.0–0.4]	0.865
Medications for side effects	7.2 [0.0–11.0]	7.5 [0.0–10.9]	7.0 [0.0–11.0]	0.913
Medications for comorbidities	2.0 [0.0–3.3]	2.0 [0.0–3.3]	2.0 [0.0–3.3]	0.917
Nonmedical costs:	224.8 [137.1–301.3]	230.2 [143.7–295.1]	216.3 [116.8–301.3]	0.659
Food/nutrition	177.9 [107.1–232.1]	180.3 [113.2–228.7]	173.1 [92.4–236.0]	0.753
Hygienic products/other utilities	50.0 [32.8–65.3]	53.1 [32.6–66.6]	47.2 [33.0–64.8]	0.34
Outpatient care	145.5 [0.0–405.1]	100.7 [0.0–356.4]	247.2 [59.7–423.7]	0.001
Medical costs	115.0 [0.0–366.4]	70.0 [0.0–314.7]	205.2 [32.8–385.5]	0.0001
Tests and medical examinations	0.0 [0–2.8]	0.0 [0.0–1.8]	0.0 [0.0–3.1]	0.097
Medications for side effects	85.3 [0.0–269.4]	46.6 [0.0–237.0]	147.2 [0.0–292.5]	0.0001
Medications for comorbidities	0.0 [0.0–29.3]	0.0 [0.0–27.0]	0.0 [0.0–45.1]	0.015
Non-traditional medicine	0.0 [0.0–37.9]	0.0 [0.0–29.3]	0.0 [0.0–29.3]	0.443
Non-medical costs	0.0 [0.0–37.9]	0.0 [0.0–37.9]	0.0 [0.0–37.9]	0.893
Subtotal: tests and consultations	3.7 [3.6–13.2]	3.7 [3.6–11.8]	3.7 [3.6–15.3]	0.019
Subtotal: medications	103.6 [14.2–335.1]	62.8 [12.3–284.2]	172.5 [27.2–356.8]	0.0001
Subtotal: medical costs	125.3 [20.3–375.5]	84.4 [19.4–326.9]	215.5 [162.2–319.9]	<0.001
Subtotal: non-medical costs	249.5 [153.0–324.9]	253.4 [162.2–319.6]	234.5 [147.2–342.7]	0.727
Indirect cost, total	1,425.7 [387.2–2393.1]	996.2 [250.4–2369.2]	1,644.3 [505.4–2,433.1]	0.022
Income loss in inpatient care	462.0 [164.6–387.3]	425.7 [96.5–630.4]	503.3 [231.8–783.3]	0.00001
Income loss in outpatient care	844.9 [462.0–1425.7]	782.1 [0.0–1765.6]	979.4 [44.4–1818.4]	0.159

IQR = interquartile range (Q2–Q3); MDL = Moldovan leu.

* Costs were calculated in MDL and converted to US dollars (exchange rate at the time of study: US$1 = MDL19.9238).

† Poverty defined using the World Bank criterion (see Table 1) and based on household income prior to TB diagnosis. Missing data were excluded during hypothesis testing.

‡ Calculated using the Mann–Whitney test.

TB-related costs in outpatient care made up 30% of the total direct costs for poor patients and 46% for non-poor TB patients, with median costs of respectively US$101 and US$247 (*P* = 0.001). In outpatient care, the largest payments made were for medications for side effects: US$84 for poor and US$216 for non-poor TB patients (*P* < 0.001).

The median direct costs incurred for diagnosis were US$1.5 per patient; these costs were the same for poor and non-poor TB patients. The median indirect costs experienced during the entire period of TB treatment were US$1,426 per TB patient: US$992 for poor patients and US$1,644 for non-poor patients (Table 3**)**. TB patients lost 30% of their total household income in inpatient care and 70% in outpatient care.

Using the catastrophic cost threshold level of ≥20%, 26% (112/430) of TB patients experienced catastrophic costs; 17% (74/430) of these were from poor households. Excluding incentives from the measurement of catastrophic costs gives the proportion of households affected by TB with catastrophic costs as 46% (197/430) of all participants, 55% (131/238) of poor TB patients, and 34% (66/192) of non-poor TB patients (*P* > 0.05) (Figure).

**Figure.**
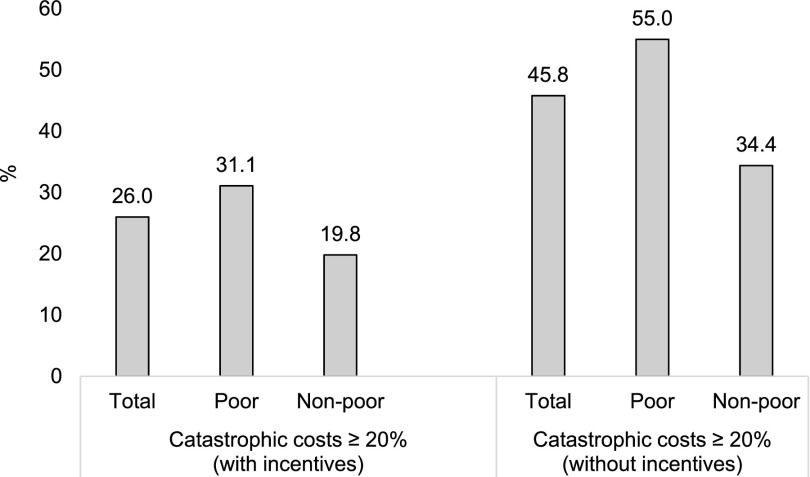
Proportion of catastrophic costs due to TB (with and without incentives) by income at household level, Republic of Moldova, 2016.^†^* *According to World Bank criterion (poor household: household earning <US$1.9 per capita per day; non-poor household: ≥US$1.9 per capita per day) and based on household income prior to TB diagnosis. Missing data were excluded during hypothesis testing. ^†^Two proportion *Z*-test (catastrophic costs with incentives, *P* < 0.01; catastrophic costs without incentives, *P* < 0.001).

Four determinants associated with catastrophic costs were identified in the adjusted analysis: being unofficially employed or unemployed (aOR 1.9, 95% CI 1.1–3.3), having fewer household members (aOR 2.1, 95% CI 1.3–3.5), having an income accounting for over 50% of total household income (aOR 2.4, 95% CI 1.5–3.8), and being a poor household (aOR 2.2, 95%CI 1.2–3.9) (Table 4).

**Table 4.** Associations between potential risk factors and catastrophic costs due to TB among patients with rifampicin-resistant TB, Republic of Moldova, 2016.

Characteristics	TB-related costs		cOR (95% CI)	aOR (95% CI)
	≤19% *n* (% row)	≥20% *n* (% row)		
Total	318 (74)	112 (26)		
Sex				
Male	254 (75.6)	82 (24.4)	Reference	Reference
Female	64 (68.1)	30 (31.9)	1.45 (0.88–2.39)	1.34 (0.77–2.33)
Age, years				
≤39	144 (72.7)	54 (27.3)	1.12 (0.73–1.73)	0.82 (0.51–1.31)
≥40	174 (75.0)	58 (25.0)	Reference	Reference
Residence				
Urban	119 (74.4)	41 (25.6)	Reference	—
Rural	199 (73.7)	71 (26.3)	1.04 (0.66–1.62)	—
Level of education				
None/primary	110 (76.9)	33 (23.1)	0.79 (0.49–1.26)	—
Secondary/vocational/higher	208 (72.5)	79 (27.5)	Reference	—
Marital status				
Married/cohabitating	176 (78.6)	48 (21.4)	Reference	Reference
Unmarried	142 (68.9)	64 (31.1)	1.65 (1.07–2.55)	1.21 (0.73–1.98)
Household size, person				
≤2	63 (56.2)	132 (41.5)	1.81 (1.17–2.8)	2.13 (1.28–3.53)
>3	49 (43.8)	186 (58.5)	Reference	Reference
Employment				
Official employed	136 (84.0)	26 (16.0)	Reference	Reference
Unofficially employed or not employed	182 (67.9)	86 (32.1)	2.47 (1.51–4.04)	1.86 (1.06–3.28)
Households in poverty*				
Poor	164 (68.9)	74 (31.1)	1.83 (1.17–2.86)	2.2 (1.24–3.89)
Non-poor	154 (80.2)	38 (19.8)	Reference	Reference
Wealth Index^†^				
Low standard of living	158 (73.5)	57 (26.5)	1.05 (0.68–1.61)	—
High standard of living	160 (74.4)	55 (25.6)	Reference	—
Patient annual income as proportion of annual household income				
≤50%	51 (45.5)	95 (29.9)	Reference	Reference
≥51%	61 (54.5)	223 (70.1)	1.96 (1.26–3.06)	2.37 (1.48–3.81)
Health insurance prior to TB				
Yes	169 (74.8)	57 (25.2)	1.09 (0.71–1.68)	—
No	149 (73.0)	55 (27.0)		—
History of migration				
Yes	63 (80.8)	15 (19.2)	0.63 (0.34–1.15)	—
No	255 (72.4)	97 (27.6)	Reference	—
History of incarceration^‡^				
Yes	28 (68.3)	13 (31.7)	1.40 (0.70–2.82)	—
No	284 (75.1)	94 (24.9)	Reference	—
Current smoking status				
Yes	190 (77.2)	56 (22.8)	0.67 (0.44–1.04)	0.67 (0.41–1.08)
No	128 (69.6)	56 (30.4)	Reference	Reference
Alcohol consumption				
Yes	31 (68.9)	14 (31.1)	1.32 (0.67–2.59)	—
No	287 (74.5)	98 (25.5)	Reference	—
Injectable/non-injectable drug use				
Yes	32 (74.4)	11 (25.6)	0.97 (0.47–2.0)	—
No	286 (73.9)	101 (26.1)	Reference	—
History of TB treatment				
New	211 (73.0)	78 (27.0)	Reference	—
Relapse	107 (75.9)	34 (24.1)	0.86 (0.54–1.37)	—
HIV				
Yes	26 (72.2)	10 (27.8)	1.10 (0.51–2.36)	—
No	292 (74.1)	102 (25.9)	Reference	—
Diabetes mellitus				
Yes	6 (54.5)	5 (45.5)	2.42 (0.57–9.75)	—
No	312 (74.5)	107 (25.5)	Reference	—
Comorbidities (other)				
Yes	91 (67.4)	44 (32.6)	1.61 (1.03–2.53)	1.49 (0.93–2.39)
No	227 (76.9)	68 (23.1)	Reference	Reference
Side effects				
Yes	258 (73.3)	94 (26.7)	1.21 (0.68–2.16)	—
No	60 (76.9)	18 (23.1)	Reference	—

* According to World Bank criterion (poor household: household earning <US$1.9 per capita per day; non-poor household: ≥US$1.9 per capita per day) and based on household income prior to TB diagnosis. Missing data were excluded during hypothesis testing.

† Standard of living categorised using the Wealth Index.

‡ Data available from 419 patients.

CI = confidence interval; cOR = crude odds ratio; aOR = adjusted OR.

## DISCUSSION

Our study provides an overview of the financial burden experienced by households affected by RR-TB in the Republic of Moldova. This is the first study conducted in the WHO European Region to quantify the catastrophic costs related to TB and to investigate the determinants of these costs. More than one quarter of households affected by RR-TB experienced catastrophic costs in the country in 2016. Studies from 2018–2019 on this topic from countries outside the European Region reported higher catastrophic costs in RR-TB households, such as 83% in Indonesia,^15^ 73% in Ghana,^16^ 63% in Viet Nam^17^ and 54% in Peru.^18^ One explanation for these different values could be differences in the geographical, health system, and economic profiles of the countries.^19^ The second explanation could be the existence of adherence incentives for TB patients in the Republic of Moldova,^20^ which could exert some protective (cost-containing) effects on the TB-associated costs of households, as observed by our results. In all these studies, the poorest patients were the most likely to incur catastrophic costs due to TB.

Long-term hospital admissions generate a loss of income for households. In our study, patients with TB lost one-third of their incomes and incurred most of their TB-related costs during their hospital stays. An association between TB-related costs and hospitalisations had previously been indicated by studies investigating TB-associated costs.^21,22^ The largest expenditure in inpatient care among the study participants was for food. Nutrition plays an important role in the healing process of TB.^23^ The significant nutrition costs incurred during hospital stays suggest inadequate nutrition provision by the health system for inpatient TB care that should be revised and adapted to the needs of RR-TB patients.

The toxicity of TB drugs administered for RR-TB treatment requires additional medication to deal with side effects; this medication is provided free of charge only during hospital stays. However, our study shows that costs for such medication were incurred by TB patients in both inpatient and outpatient TB care. The study also underlines that poor patients spend less than non-poor TB patients on these medicines in outpatient care, indicating that poorer TB patients may not be able to afford the costs of these medicines, especially since approximately 70% of income is lost during outpatient treatment. This highlights the necessity of developing a method to provide the public health sector with this medication for patients receiving outpatient TB care.

Several sociodemographic factors that could lead to TB catastrophic costs were identified by this study, such as being unofficially employed or unemployed, having fewer household members, the TB patient having an income that accounted for over 50% of the total household income, and poverty.

Unemployment or being unofficially employed could influence healthcare costs. An association between catastrophic TB costs and unemployment has been observed in studies conducted in Peru,^18^ South Africa,^24^ and Kenya.^25^ Often, for people who are unofficially employed, employers do not take responsibility for covering the temporary disability allowance for TB, which is fully covered for people who are in official employment.^26^ In the Republic of Moldova, government wages, social pensions, and other unemployment benefits are low; therefore, people look for informal work or work abroad.^27,28^

Living alone or in a small household was identified as a determinant of catastrophic TB costs, and this association was also observed in South Africa,^24^ Nigeria,^29^ and Malawi.^30^ Therefore, in households with many members, there are more income earners, and the impact when one earner falls ill with TB is reduced. Research in Indonesia observed that people in low-income households not only have higher risks of getting TB, but once they are infected, high TB-related costs may reduce them to poverty.^31^ Our results showed that households experienced TB-related costs while their incomes were shrinking, thus contributing to a ‘medical poverty trap’.^32,33^ The annual income of patients with TB included in our study was 1.5 times less than the Moldovan gross domestic product per capita in 2016.^34^

The key components of the WHO’s End TB Strategy 2016–2035^2^ include reducing poverty, advocating improved equity of access to health care, universal health care, and eliminating catastrophic costs in households affected by TB. In this context, it is apparent that TB is no longer only a communicable disease issue but is rather a complex socioeconomic phenomenon, with TB control interventions requiring a broader approach, including the targeting of the economic effects of the disease.

## CONCLUSIONS

One quarter of RR-TB patients faced catastrophic costs due to TB despite universal access to TB diagnosis and treatment in the Republic of Moldova. TB-related costs were mostly incurred through income loss and payments for nonmedical products in inpatient care, and there was inequity in the distribution of TB-related costs in inpatient and outpatient care. Several risk factors that affected the occurrence of catastrophic costs were identified and should be considered when designing policies for the model of care for patients with RR-TB to limit the risk of catastrophic costs in selected vulnerable groups.

In context, TB control requires updating patient-centred TB improvement strategies and mitigating key barriers to accessing care. These findings outline the potential role of social protection not only as a poverty reduction strategy but also as a tool for better disease control, with a trickle-down effect on health.
